# Evidence for a role of angiotensin converting enzyme 2 in proteinuria of idiopathic nephrotic syndrome

**DOI:** 10.1042/BSR20181361

**Published:** 2019-01-03

**Authors:** Roberta da Silva Filha, Sérgio Veloso Brant Pinheiro, Thiago Macedo e Cordeiro, Victor Feracin, Érica Leandro Marciano Vieira, Aline Silva Miranda, Ana Cristina Simões e Silva

**Affiliations:** 1 Interdisciplinary Laboratory of Medical Investigation, Faculty of Medicine, Universidade Federal de Minas Gerais (UFMG), Brazil; 2 Unit of Pediatric Nephrology, Department of Pediatrics, Faculty of Medicine, UFMG, Brazil; 3 Laboratory of Neurobiology, Department of Morphology, Institute of Biological Sciences, UFMG, Brazil

**Keywords:** cytokines, nephrotic syndrome, proteinuria, renin-angiotensin system

## Abstract

**Introduction:** Renin angiotensin system (RAS) plays a role in idiopathic nephrotic syndrome (INS). Most studies investigated only the classical RAS axis. Therefore, the aims of the present study were to evaluate urinary levels of RAS molecules related to classical and to counter-regulatory axes in pediatric patients with INS, to compare the measurements with levels in healthy controls and to search for associations with inflammatory molecules, proteinuria and disease treatment. **Subjects and methods:** This cross-sectional study included 31 patients with INS and 19 healthy controls, matched for age and sex. Patients and controls were submitted to urine collection for measurement of RAS molecules [Ang II, Ang-(1-7), ACE and ACE2] by enzyme immunoassay and cytokines by Cytometric Bead Array. Findings in INS patients were compared according to proteinuria: absent (<150 mg/dl, *n* = 15) and present (≥150 mg/dl, *n* = 16). **Results:** In comparison to controls, INS patients had increased Ang II, Ang-(1-7) and ACE, levels while ACE2 was reduced. INS patients with proteinuria had lower levels of ACE2 than those without proteinuria. ACE2 levels were negatively correlated with 24-h-proteinuria. Urinary concentrations of MCP-1/CCL2 were significantly higher in INS patients, positively correlated with Ang II and negatively with Ang-(1-7). ACE2 concentrations were negatively correlated with IP-10/CXCL-10 levels, which, in turn, were positively correlated with 24-h-proteinuria. **Conclusion:** INS patients exhibited changes in RAS molecules and in chemokines. Proteinuria was associated with low levels of ACE2 and high levels of inflammatory molecules.

## Introduction

The pathophysiological mechanisms of idiopathic nephrotic syndrome (INS) support an underlying role for the immune system [1,2]. This hypothesis relies on the fact that the main treatment is based on corticosteroids and studies show different inflammatory profiles in patients with INS [3–6]. Experimental and clinical studies also suggest that renin angiotensin system (RAS) may play an important role on the pathogenesis of INS [7–10].

RAS is classically described as a circulating system that leads to the production of angiotensin II (Ang II), which binds to angiotensin type 1 receptors (AT_1_), promoting vasoconstriction, sodium retention, aldosterone release, inflammation and fibrosis [11]. However, RAS is now conceived as a dual acting system, mainly formed by two opposite axes [12–14] the classical one, including angiotensin converting enzyme (ACE), Ang II and AT_1_ receptor and the counter-regulatory axis formed by the enzyme homologue to ACE, named ACE2 [15,16], the heptapeptide Angiotensin-(1-7) [Ang-(1-7)] and its receptor, Mas [17]. Ang-(1-7) is mainly produced by ACE2 that uses Ang II as the major substrate [18]. Ang-(1-7) exerts its effects via Mas receptor activation, leading to vasodilation, anti-inflammatory and anti-fibrogenic effects [12–14]. The unbalance between both RAS axes is thought to have an active role in renal diseases [12].

Experimental studies indicate that activation of the counter-regulatory RAS axis reduces renal inflammation, fibrosis and proteinuria. In a murine model of nephrotic syndrome (NS), the treatment with a Mas receptor agonist, the compound AVE0991, reduced proteinuria, renal levels of TGF-beta and renal tissue damage [8]. On the other hand, NS induced in Mas receptor knockout mice did not improved in response to the use of AT_1_ receptor blocker (ARB), suggesting that the presence of Mas receptor is critical for the therapeutic response to ARBs [8]. Despite clinical studies showing beneficial effects of ACE inhibitors and ARBs [6,7], there are no studies evaluating the role of the counter-regulatory RAS axis in INS patients, mostly in pediatric population. Therefore, the aims of the present study were to evaluate urinary levels of RAS molecules [(ACE, ACE2, Ang II and Ang-(1-7)] in pediatric patients with INS and to compare with the same measurements in healthy sex- and age-matched children. In addition, measurements of RAS molecules were correlated with proteinuria and with urinary levels of markers of inflammation in pediatric patients with INS.

## Subjects and methods

### Study design

This is a cross-sectional study with a sample of children and adolescents with remitted and partially remitted INS, followed-up at the Pediatric Nephrology Unit (PNU) of our institution from 2017 to 2018. Our PNU has attended approximately 300 children with nephrotic syndrome, according to a systematic protocol that includes definition of disease etiology, assessment of clinical course and laboratory alterations, institution of treatment protocols and indication of renal biopsy based on clinical (corticosteroid unresponsiveness) and laboratory findings as detailed elsewhere [19]. The diagnostic criteria for INS were based on the KDIGO Clinical Practice Guideline for Glomerulonephritis (2012) [20].

### Patients with INS

Thirty-one patients ranging from 8 to 16 years with INS in total or partial remission were included in the study. The inclusion criteria were still-preserved renal function, well-established diagnosis of INS and complete or partial remission of the disease. After parents’ consent, urine samples were collected simultaneously to routine laboratory exams. Exclusion criteria were congenital nephrotic syndrome, secondary forms of nephrotic syndrome, INS patients at stages 2–5 of chronic kidney disease, INS patients during clinical and laboratory relapses and the presence of acute infections and allergies at the moment of urine collection.

### Control group

The control group consisted of 19 healthy sex- and age-matched subjects from the Pediatric Primary Care Center. Healthy status was determined through the subjects’ medical history and either a parental report or self-report to rule out the presence of chronic or acute diseases.

### Ethical aspects

The Ethics Committee of our institution approved the present study under the protocol CAAE-07513513.9.0000.5149. Informed consent was obtained from the parents of all included subjects. The research protocol did not interfere with any medical recommendations or prescriptions. The follow-up of the INS patients and healthy controls was guaranteed even in cases of refusal to participate in the study.

### Study protocol

The present study included only patients presenting complete or partial remission of INS. Patients with 24-h urine protein excretion above or equal to 150 mg/dl and absence of edema were considered in partial remission. Patients with 24 h urine protein excretion below 150 mg/dl and absence of edema were considered in complete remission. Patients exhibiting relapses of edema and intense proteinuria were excluded. The established reference for proteinuria was based on the KDIGO [20]. Clinical characteristics and casual measurements were obtained at the same time of urine collection. Clinical variables were age, gender, height, weight, body mass index, and systolic and diastolic blood pressure. In INS patients, serum levels of creatinine, albumin, cholesterol and triglycerides were assessed at the same time of urine collection obtained for the measurements of RAS molecules and immune mediators. Urinary determinations of creatinine and of 24-h protein excretion were also performed simultaneously to other measurements. Glomerular filtration rate (GFR) was estimated using the modified formula of Schwartz et al. [21]. Renal biopsy results and medications in use at the time of urine sampling were also analyzed.

INS patients were subdivided according to the values of proteinuria, which ranged from 48 mg/dl to 1220 mg/dl. Thereby, those with 24-h urinary protein excretion inferior to 150 mg/dl were allocated to the subgroup named absence of proteinuria (*n* = 15) and when proteinuria was equal to or above 150 mg/dl patients were included in the subgroup called presence of proteinuria (*n* = 16).

INS patients were also analyzed in regard to the use or not of medications that directly interfere with RAS as ACE inhibitors and/or ARBs and to the use or not of steroids. Therefore, 16 patients in use of ACE inhibitors and/ or ARBs were compared with 15 patients not receiving these medications and 18 patients in use of steroids were compared with 13 not under treatment at the time of urine sampling.

### Urine samples

Urine specimens for the measurement of biomarkers were collected into sterile dry tubes. After homogenization, 15 ml of the collected urine were centrifuged at 4°C for 5 min and aliquoted into 1 ml tubes and stored at −80°C until the measurements.

### Renin angiotensin system (RAS) components

Urine levels of RAS molecules [Ang II, Ang-(1-7), ACE and ACE2] were measured by enzyme immunoassay (ELISA), according to procedures supplied by the manufacturer (MyBioSource, San Diego, CA, USA). All kits applied sandwich ELISA technique, except for ACE measurement whose kit applied competitive ELISA method. The sensitivity of the assays was 1.0 pg/ml for ACE and ACE2, 2.0 pg/ml for Ang II and Ang-(1-7) and reading the optical density at 450 nm. All biochemical assessments were performed blinded in regard to clinical diagnosis.

### Cytokines and chemokines measurements

The urinary levels of multiple cytokines [interleukin (IL)-12p70, IL-6, IL-8, IL-10, IL-1β, tumor necrosis factor (TNF) and interferon gamma (IFN-γ)] and chemokines [induced protein 10 (IP-10/CXCL-10), monocyte chemoattractant protein-1 (MCP-1/CCL2), IL-8/CXCL8, monokine induced by gamma interferon (MIG/CXCL9), regulated on activation normal T cell expressed and secreted (RANTES/CCL5)] were assessed simultaneously using a Human FlexSet kit for Cytometric Bead Array (CBA, BD Bioscience, San Jose, CA, USA), following manufacture’s instruction. The acquisition was performed using an FACSCanto II flow cytometer (BD Biosciences, San Jose, CA, USA). The instrument has been checked for sensitivity and overall performance with Cytometer Setup & Tracking beads (BD Biosciences) prior to data acquisition. Quantitative results were generated using FCAP Array v1.0.1 software (Soft Flow Inc., Pecs, Hungary). Urinary levels of all these biomarkers were expressed as concentrations standardized for urine creatinine and expressed as pictograms per milligram. Positive controls were also included in urine measurements of cytokines and chemokines to confirm the accuracy of the assays.

### Statistical analysis

The softwares SPSS version 22.0 (SPSS Inc., Chicago, IL, USA) and GraphPad Prism 5.0 (GraphPad Software, Inc., La Jolla, CA, USA) were used for statistical analysis. The results obtained were expressed as means and standard error of mean (SEM), medians and interquartile range or percentages, when appropriate. Categorical variables were compared by Qui-square. Gaussian distribution was checked by Shapiro–Wilk test. For variables without Gaussian distribution, Mann–Whitney test was used to compare two groups. For variables with normal distribution, comparisons between two groups were made by unpaired Student’s *t* test. Spearman’s correlation analyses examined the relationship between proteinuria, urinary levels of RAS components and measurements of inflammatory molecules in the same samples. All statistical tests were two-tailed with a significance level of *P* < 0.05.

## Results

### Subject characteristics and casual measurements

Clinical and laboratory findings of INS patients and healthy controls are shown in Table 1. The control group (*n* = 19) included 12 boys and 7 girls, ranging from 9 to 15 years old. All controls had clinical and laboratory parameters within normal range (Table 1). No statistical differences between INS patients (*n* = 31) and controls were found in age, sex distribution, body mass index, GFR, plasma levels of triglycerides, cholesterol, creatinine and albumin (*P* > 0.05 for all comparisons, Table 1). As expected, the values of proteinuria were significantly higher in INS patients than in healthy controls (Table 1).

**Table 1:** Clinical and laboratorial findings of patients with idiopathic nephrotic syndrome and healthy controls matched by sex and age

Parameters	Patients (*n* = 31)	Controls (*n* = 19)	*P* values
Age (years)	11.3 ± 4.8	11.9 ± 1.8	0.912
Sex (male / female)	19 / 12	12 / 7	0.905
BMI (kg/m^2^)	19.3 ± 3.2	17.8 ± 2.6	0.094
Creatinine (mg/dl)	0.6 ± 0.2	0.6 ± 0.1	0.210
Triglycerides (mg/dl)	114.4 ± 79.6	–	–
Total cholesterol (mg/dl)	188.8 ± 92.9	–	–
Albumin (g/dl)	4.0 ± 0.7	4.5 ± 0.2	
Glomerular filtration rate^*^	109.2 ± 28.8	119 ± 12	0.254
Proteinuria (mg/m^2^/24 h)	304.2 ± 378.5	<100 mg/dl	–
Medications in use			–
No medication	4	19	*<0.001*
Only steroids	2	–	–
Steroids + ACEi or ARB	16	–	–
Only ACEi or ARB	0	–	–
Only cyclosporine	0	–	–
Cyclosporine + ACEi or ARB	9	–	–
Histopathology			
No biopsy	15	–	–
MDC	5	–	–
FSGS	5	–	–
Membranous nephropathy	3	–	–
Mesangial glomerulopathy	3	–	–

Values are expressed as mean and standard deviation. Sex, medications in use and renal histopathology are expressed as absolute values.

*Glomerular filtration rate was estimated using modified Schwartz’s formula. Abbreviations: ACEi, inhibitor of the angiotensin converting enzyme; ARB, angiotensin receptor type 1 blocker; BMI, body mass index; FSGS, focal segmental glomerulosclerosis; MDC, minimal change disease.

In regard to treatment, 6 among 31 (19%) INS patients were not receiving any medication at the time of urine collection, whereas the remaining patients needed at least one medication (Table 1). Sixteen among 25 (64%) patients of the INS group were receiving steroids and 9 (36%) cyclosporine, both isolated or in association with inhibitors of the RAS. In regard to histopathology, 15 (48.4%) patients were not submitted to renal biopsy, 5 (16.1%) presented focal segmental glomerulosclerosis, 5 (16.1%) had minimal change disease, 3 (9.7%) exhibited mesangial glomerulopathy and 3 (9.7%) membranous glomerulopathy.

The comparison between INS patients without proteinuria (absent) versus patients with proteinuria (present) is shown in Table 2. No differences in clinical and laboratorial findings were detected (*P* > 0.05 for all comparisons, Table 2), except for 24-h proteinuria.

**Table 2:** Clinical and laboratorial findings of patients with idiopathic nephrotic syndrome subdivided according to 24-h urinary protein excretion: absence of proteinuria ≤150 mg/dl (without proteinuria) and presence of proteinuria >150 mg/dl (with proteinuria)

Parameters	Without proteinuria (*n* = 15)	With proteinuria (*n* = 16)	*P* values
Age (years)	10.6 ± 3.2	13.1 ± 4.4	0.385
Sex (male / female)	10 / 5	7 / 7	0.078
BMI (kg/m^2^)	19.1 ± 2.5	19.7 ± 3.9	0.981
Creatinine (mg/dl)	0.53 ± 0.10	0.70 ± 0.32	0.162
Triglycerides (mg/dl)	98.5 ± 59.7	132.7 ± 97.1	0.205
Total cholesterol (mg/dl)	186.1 ± 107.8	186.4 ± 78.3	0.519
Albumin (g/dl)	4.1 ± 0.76	3.9 ± 0.5	0.285
Glomerular filtration rate^*^	113.8 ± 25.7	102.1 ± 32.8	0.326
Proteinuria (mg/m^2^/24 h)	80.7 ± 25.4	543.7 ± 434.1	***<0.0001***
Medications in use			
Only steroids	1	1	0.859
Steroids + ACEi or ARB	4	10	0.076
Only ACEi or ARB	0	0	0.125
Only cyclosporine	0	0	0.806
Cyclosporine + ACEi or ARB	5	4	0.806
No medications	5	1	0.093
Histopathology			0.535
No biopsy	6	7	
MDC	4	1	
FSGS	2	3	
Membranous nephropathy	1	2	
Mesangial glomerulopathy	2	1	

Values are expressed as mean and standard deviation. Sex, medications and renal histopathology in use are expressed as absolute values.

*Glomerular filtration rate was estimated using modified Schwartz’s formula. Abbreviations: ACEi, inhibitor of the angiotensin converting enzyme; ARB, angiotensin receptor type 1 blocker; BMI, body mass index; FSGS, focal segmental glomerulosclerosis; MDC, minimal change disease.

Table 3 shows the comparison between patients receiving (*n* = 16) and not receiving ACE inhibitors and/or ARBs (*n* = 15). The only difference between both subgroups was the medication in use. No significant differences were detected in regard to age, sex distribution, clinical data, laboratory measurements and renal histopathology (*P* > 0.05 for all comparisons, Table 3).

**Table 3:** Clinical and laboratorial findings of patients with idiopathic nephrotic syndrome subdivided according to use or not of medications that directly interfere with RAS: ACE inhibitors (ACEi) and ARB.

Parameters	ACEi or ARB (*n* = 25)	No ACEi or ARB (*n* = 6)	*P* values
Age (years)	11.4 ± 4.6	10.7 ± 4.5	0.585
Sex (male / female)	14 / 9	5 / 3	0.957
BMI (kg/m^2^)	19.8 ± 3.3	13.9 ± 8.8	0.260
Creatinine (mg/dl)	0.61 ± 0.28	0.54 ± 0.12	0.964
Triglycerides (mg/dl)	117.9 ± 87.2	103.9 ± 55.9	0.852
Total cholesterol (mg/dl)	186.2 ± 68.8	197 ± 153.8	0.320
Albumin (g/dl)	3.9 ± 0.5	4.2 ± 0.9	0.181
Glomerular filtration rate^*^	108.1 ± 29.4	112.3 ± 28.6	0.910
Proteinuria (mg/m^2^/24 h)	380.2 ± 421.8	104.9 ± 44.6	0.118
Medications in use		–	
Only steroids	0	2	*0.032*
Steroids + ACEi or ARB	16	0	–
Only ACEi or ARB	0	0	–
Only cyclosporine	0	0	–
Cyclosporine + ACEi or ARB	9	0	–
No medications	0	4	*<0.0001*
Histopatology		0.920	
No biopsy	10	5	
MDC	4	0	
FSGS	4	1	
Membranous nephropathy	2	1	
Mesangial glomerulopathy	2	1	

Values are expressed as mean and standard deviation. Sex, medications and renal histopathology in use are expressed as absolute values.

*Glomerular filtration rate was estimated using modified Schwartz’s formula. Abbreviations: ACEi, inhibitor of the angiotensin converting enzyme; ARB, angiotensin receptor type 1 blocker; BMI, body mass index; FSGS, focal segmental glomerulosclerosis; MDC, minimal change disease.

### RAS components analyses

**Figure 1:**
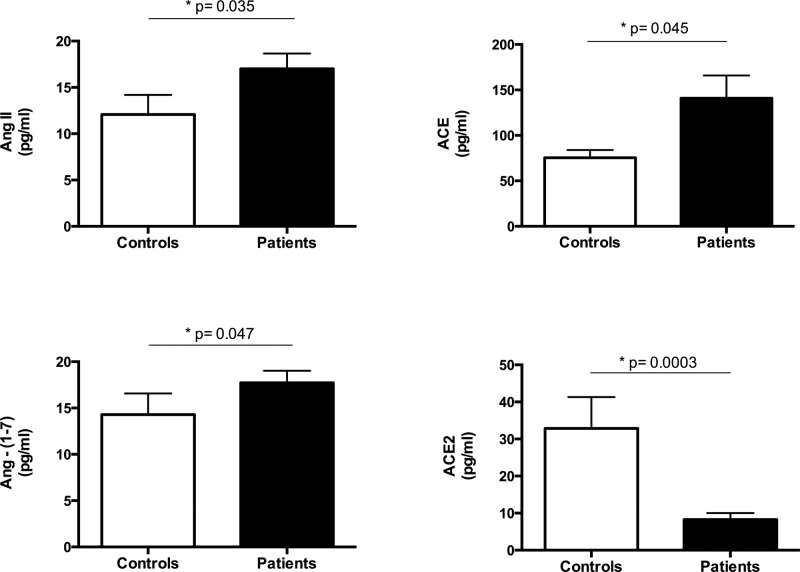
Levels of RAS components in urine samples of patients with INS and healthy sex and age-matched subjects (controls) Patients with INS presented increased urinary levels of angiotensin (Ang) II, angiotensin-converting enzyme (ACE) and Ang-(1-7) in comparison with controls. On the other hand, ACE2 levels were lower in INS patients than in controls. Results are expressed as bar graphs with mean values and SEM. Differences were considered to be significant at *P* < 0.05 (Mann–Whitney *U* test).

In comparison to healthy controls, INS patients presented increased urinary levels of Ang II, Ang-(1-7) and ACE (*P* < 0.05 for all comparisons, Figure 1). On the other hand, urinary levels of ACE2 were significantly lower in INS patients than in controls (*P* < 0.0003, Figure 1).

Correlations were evaluated between RAS components and proteinuria. In INS patients, urinary levels of ACE2 were negatively correlated with 24-urine protein excretion (rho = −0.508, *P* = 0.005). Furthermore, INS patients with proteinuria had significantly lower levels of ACE2 in urine than INS patients without proteinuria (*P* = 0.02, Table 4). Regarding other RAS components [Ang II, ACE and Ang-(1-7)], there were no correlations with 24-urine protein excretion. Accordingly, no differences were found in the comparison of these molecules in INS patients without proteinuria versus those with proteinuria (Table 4).

**Table 4:** Urinary levels of RAS components in idiopathic nephrotic syndrome patients without proteinuria (≤150 mg/dl/day) and with proteinuria (>150 mg/dl/day)

RAS components	Without proteinuria (*n* = 15)	With proteinuria (*n* = 16)	*P* values
Ang II (pg/ml)	16.4 ± 8.4	14.7 ± 8.0	0.570
ACE (pg/ml)	98.4 ± 50.0	172.9 ± 162.2	0.247
Ang-(1-7) (pg/ml)	17.5 ± 7.5	16.0 ± 6.8	0.662
ACE2 (pg/ml)	13.5 ±10.7	2.9 ± 5.2	***0.023***

Values are expressed as mean ± standard deviation. Abbreviations: ACE, angiotensin converting enzyme; ACE2, angiotensin converting enzyme 2; Ang II, angiotensin II; Ang-(1-7), angiotensin-(1-7).

### Chemokines and cytokines analyses

**Figure 2:**
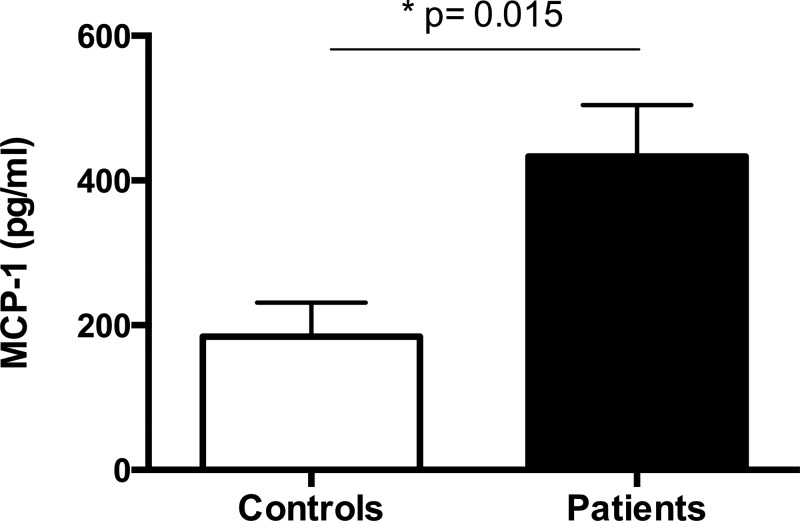
Urinary levels of MCP-1/CCL2 in patients with primary nephrotic syndrome (INS) and in healthy sex and age-matched subjects (controls) Patients with INS had increased urinary levels of MCP-1/CCL2. Results are expressed as bar graphs with mean values and SEM. Differences are considered to be significant at *P* < 0.05 (Mann–Whitney *U* test). MCP-1/CCL2 = monocyte chemoattractant protein-1.

As shown in Figure 2, urinary concentrations of MCP-1/CCL2 were significantly higher in INS patients when compared with the control group (*P* = 0.015). However, urinary levels of other chemokines (IP-10/CXCL-10, MIG/CXCL9, RANTES/CCL5, IL-8/CCCL8) and of cytokines (IL-12p70, TNF, IL-10, IL-6, IL-1β, IFN) did not significantly differ in the comparison of INS patients and healthy controls.

We also investigated the relation between urinary levels of chemokines and cytokines with 24-h urine protein excretion. Urinary levels of IP-10/CXCL-10 were positively correlated with 24-h urine protein excretion in INS patients (rho = 0.3882, *P* = 0.037). No correlations were found between 24-h urine protein excretion with other chemokines and cytokines.

### Correlations between RAS components and immune system molecules in INS patients

**Figure 3:**
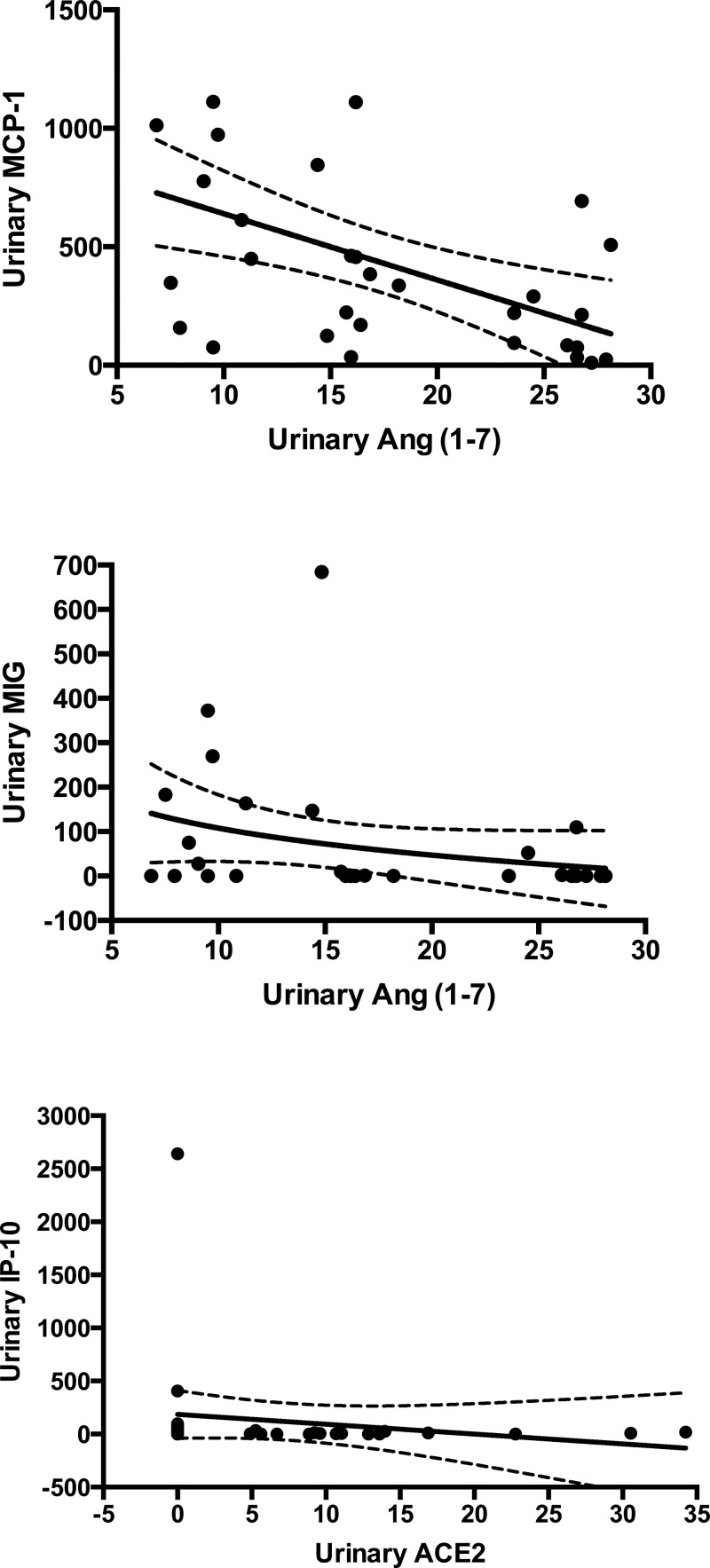
Correlations between RAS molecules and chemokines Panel superior shows a negative correlation between urinary levels of Angiotensin-(1-7) [Ang-(1-7)] and urinary levels of MCP-1/CCL2 (rho = −0.485, *P* = 0.006, Spearman correlation test). Panel intermediate displays a negative correlation between urinary levels of Ang-(1-7) and urinary levels of and MIG (rho = −0.379, *P* = 0.035, Spearman correlation test). Panel inferior shows a negative correlation between urinary levels of Angiotensin Converting Enzyme 2 (ACE2) and urinary levels of IP-10/CXCL-10 (rho = −0.420, *P* = 0.018, Spearman correlation test).

Urinary levels of chemokines and cytokines were also checked for correlations with RAS molecules in the same urine samples. In INS patients, levels of Ang II positively correlated with MCP-1/CCL2 levels (rho = 0.424, *P* = 0.017). On the other hand, as shown in Figure 3, Ang-(1-7) levels negatively correlated with MCP-1/CCL2 (rho = −0.485, *P* = 0.006, Figure 3 – panel superior) and MIG (rho = −0.379, *P* = 0.035, Figure 3 – panel intermediate) and ACE2 levels were negatively correlated with IP-10/CXCL-10 (rho = −0.420, *P* = 0.018, Figure 3 – panel inferior). No correlations were found between cytokines and RAS molecules.

### Effect of RAS inhibition on RAS components, proteinuria and immune system molecules

As shown in Table 3, 16 patients were receiving ACE inhibitors or ARBs at the time of urine collection. No differences were detected in urine levels of RAS molecules and in proteinuria when INS patients receiving RAS inhibitors were compared with those not receiving these medications. The same profile was not observed when inflammatory molecules were analyzed. INS patients receiving ACE inhibitors or ARBs had significantly higher urinary levels of IP-10/CXCL-10 and of IL-10 in comparison to patients not under RAS inhibition (Table 5).

**Table 5:** Urinary levels of inflammatory molecules in patients with idiophatic nephrotic syndrome subdivided according to the use or not of medications that directly interfere with RAS: ACEi and ARBs.

Inflammatory molecules	Use ACEi and/or ARB (*n* = 16/11)	No use ACEi and/or ARB (*n* = 15/20)	*P* values
IP-10 (pg/ml)	134.8 ± 506.9	98.0 ± 448.1	*0.008*
MCP-1 (pg/ml)	454.8 ± 406.9	416.3 ± 384.5	0.675
MIG (pg/ml)	83.7 ± 158.6	55.3 ± 134.6	0.115
IL-8 (pg/ml)	26.7 ± 71.3	23.2 ± 64.6	0.187
IL-12p70 (pg/ml)	9.6 ± 9.0	6.7 ± 8.3	0.083
TNF (pg/ml)	10.6 ± 10.3	7.4 ± 9.3	0.095
IL-10 (pg/ml)	7.0 ± 6.7	4.6 ± 6.1	*0.042*
IL-6 (pg/ml)	12.1 ± 9.7	9.3 ± 9.5	0.056
IL-1β (pg/ml)	26.1 ± 22.6	18.4 ± 20.1	0.219

Values are expressed as mean ± standard deviation.

### Effect of steroids administration on RAS components and immune system molecules

INS patients were also subdivided in regard to the use or not of steroids at the time of urinary measurements of RAS components, cytokines and chemokines. Urinary levels of ACE2 were significantly lower in patients under steroid therapy in comparison to those not receiving this medication (3.6 ± 4.7 pg/ml versus 15.2 ± 9.3 pg/ml, *P* = 0.033). However, urinary levels of ACE, Ang II and Ang-(1-7) did not significantly differ in these subgroups. In regard to cytokines and chemokines, patients receiving steroids had higher urinary levels of IP-10/CXCL-10 (128.6 ± 81.5 pg/ml versus 95.7 ± 125.7 pg/ml, *P* = 0.016) than those not under this treatment at the time of urine sampling. Other molecules did not significantly differ in these subgroups.

## Discussion

INS patients in complete or partial remission had significantly higher levels of ACE, Ang II and Ang-(1-7) in urine, while urinary concentrations of ACE2 were significantly lower than in healthy controls. In addition, ACE2 levels were significantly reduced in INS patients with proteinuria in comparison to those without proteinuria and urinary concentrations of this enzyme negatively correlated with 24-h urinary protein excretion.

Acquired or genetic deficiency of ACE2 exacerbated kidney injury and proteinuria in many experimental models of renal diseases, possibly facilitating the deleterious effects of Ang II [22–27]. Renal expression of ACE2 was reduced in renal cortex of mice submitted to 5/6 nephrectomy and in a rat model of renal ischemia/reperfusion [27,28]. In a model of unilateral ureteral obstruction, the deletion of ACE2 gene resulted in a four-fold increase in the ratio of intrarenal Ang II/Ang-(1-7) and these changes were associated with tubulointerstitial fibrosis and high levels of TNF, IL-1β and MCP-1 [29]. Accordingly, we found that urinary concentrations of Ang II were elevated in INS patients when compared with healthy controls and were positively correlated with urinary levels of MCP-1. More recently, the daily administration of ethanol to pregnant rats resulted in glomerulosclerosis and interstitial fibrosis of the kidneys of adult offspring, accompanied by elevated levels of serum creatinine, proteinuria, total cholesterol and reduced concentrations of serum albumin [10]. These renal alterations compatible to NS were associated with increased serum levels of Ang II, high gene expression of ACE in renal tissue and reduced expression of ACE2 and of Mas receptor in the kidneys [10]. Taken together, these experimental studies indicate that the deficiency of ACE2 promotes renal tissue lesion.

Most data regarding RAS components are obtained in experimental models. Few data from human samples corroborate our findings. Mizuiri et al. [30] showed that renal biopsies from patients with IgA nephropathy had significantly reduced glomerular and tubulointerstitial immunostaining for ACE2 when compared with healthy controls, while glomerular ACE staining was increased. These findings raise the possibility that an upward shift in the intrarenal ACE/ACE2 ratio favoring increased synthesis of Ang II and reduction in Ang-(1-7) might lead to progressive nephron loss in this condition [30]. Our research group recently detected lower urinary levels of ACE2 in children with sickle cell anemia (SCA) presenting persistent proteinuria in comparison to SCA patients with normal albumin excretion in urine, also suggesting a role of reduced ACE2 protein in renal tissue in the emergence of proteinuria and nephropathy [31].

Another interesting finding of the present study was the elevation of Ang-(1-7) levels in urine of INS patients in comparison to controls. Experimental models of renal diseases showed a protective role for Ang-(1-7) [8,29,32–38]. In an experimental model of NS, the adriamycin-induced nephropathy, oral administration of AVE 0991, a Mas receptor agonist, improved renal function, reduced proteinuria and attenuated histological changes [8]. AVE 0991 or Ang-(1-7) administration also exerted renoprotective effects in experimental acute renal injury [35] and in chronic intermittent hypoxia [38]. These effects seem to be mediated, at least in part, by reducing inflammation, oxidative stress and fibrosis [38]. The infusion of Ang-(1-7) also prevented renal lesion in a model of unilateral ureteral obstruction by suppressing renal apoptosis, fibrosis, and possibly AT_1_ receptor expression [29]. On the other hand, Ang-(1-7) administration increased ACE2 expression [29]. We may speculate that the increase in urinary levels of Ang-(1-7) would be a compensatory response to renal damage elicited by the activation of ACE-Ang II-AT_1_ receptor axis in INS patients. A similar counter-regulatory response was reported in urine of fetuses with posterior urethral valves [39]. The urine of these fetuses showed intense increase in several cytokines and chemokines [40] and these changes may be opposed by a compensatory activation of ACE2-Ang-(1-7)-Mas receptor axis [39]. Another possible explanation for urinary elevation of Ang-(1-7) could be a dysfunction or reduced expression of Mas receptor at kidney tissue. Accordingly, Ng et al. [41] reported that Mas receptor expression is reduced in the kidneys of CKD rats.

In INS patients, inflammatory molecules may also contribute to disease activity and to RAS molecules changes. Urinary concentrations of MCP-1/CCL2 were significantly higher in INS patients when compared with control group, whereas levels of IP-10/CXCL-10 in urine were positively correlated with 24-hour proteinuria. Accordingly, Vianna and co-workers [42] previously found higher urinary levels of MCP-1/CCL2 in patients with CKD due to focal segmental glomerulosclerosis than in cases of congenital uropathies. More recently, Matsumoto and co-workers [9] showed that urinary level of MCP-1/CCL2 was significantly higher in steroid resistant INS than in steroid sensitive patients, supporting the idea that urinary MCP-1/CCL2 might contribute to the recruitment of macrophages into glomeruli. Remarkably, we found that urinary concentrations of Ang-(1-7) were negatively correlated with MCP-1/CCL2, whereas ACE2 levels negatively correlated with IP-10/CXCL-10. Therefore, we believe that Ang-(1-7) may oppose the glomerular damage induced by MCP-1/CCL2 attracted macrophages. In turn, the reduced expression of ACE2 might result in altered release of IP-10/CXCL-10 in renal tissue, which affected glomerular permeability leading to proteinuria. To corroborate this hypothesis, Han et al. [43] previously reported that rats submitted to puromycin-induced nephropathy and to anti-nephrin antibody-induced nephropathy when treated with anti-IP-10/CXCL-10 function-blocking antibody displayed a decrease in the protein level of slit-diaphragm components and exacerbated proteinuria [43]. The authors also showed that the expression of CXCR3 increases in the injured podocyte in parallel with that of IP-10/CXCL-10, suggesting that IP-10 binds to CXCR3 in podocytes. The final conclusion is that IP-10/CXCL-10 may become a possible therapeutic target candidate in podocyte injury [43].

ACE inhibitors and ARBs exert renoprotective effects in glomerular diseases, mostly by reducing proteinuria [44]. However, scarce studies have investigated the effects of these medications in pediatric patients with INS [6,7]. It has also been suggested that the beneficial effects of these medications may be due, at least in part, to an activation of ACE2/Ang-(1-7)/Mas axis [45–47]. However, we did not find changes in urine concentrations of RAS components in INS patients receiving ACE inhibitors or ARBs. On the other hand, patients under treatment had higher levels of IP-10/CXCL-10 and IL-10. We did not know if changes in these cytokines are a consequence of the treatment or, alternatively, if increased levels of these molecules are related to INS itself. As already mentioned, experimental data support a role for IP-10/CXCL-10 in slit-diaphragm function [43], which might be altered in patients with more intense proteinuria. In turn, IL-10 is considered an anti-inflammatory cytokine, which may contribute to the beneficial effects of RAS blockers [48].

Our study has some limitations. First, the cross-sectional design precludes any conclusion regarding the cause of molecular changes. We probably would have obtained more conclusive results with longitudinal urine collections. Secondly, the use of immunosuppressive medications and/or RAS blockers at the time of urine collection may interfere with molecular measurements. Thirdly, we did not investigate the expression of Mas and AT_1_ receptors in renal tissue of INS patients. Fourthly, we did not use the traditional method, the radioimmunoassay, to measure RAS components. On the other hand, the inclusion of only relatively well controlled INS patients, exhibiting partial or total remission of the proteinuria, may increase the homogeneity of our sample and the results may represent more accurately changes related to disease itself rather than acute alterations due to relapses. Moreover, the utilization of a well-established protocol for molecular measurements may increase the strength of our findings [31,39,40].

In conclusion, our results support that ACE2 may exert renoprotective effects. Accordingly, genetic deficiency of ACE2 activity in mice fosters oxidative stress via AT_1_ dependent effect in the kidney [49]. In addition, daily treatment with recombinant ACE2 ameliorated renal fibrosis in apolipoprotein E-deficient mice via augmentation of Ang-(1-7)/Ang II ratio [50]. Taken together, studies indicated that the reduction in ACE2 levels at renal tissue might play a role in proteinuria and renal damage in glomerular diseases.

## Clinical perspectives

In view of the advances of INS in the last years in the pediatric population and the lack of knowledge on pathophysiological mechanisms, different hypotheses have been suggested. There is evidence that RAS molecules and changes in inflammatory and cytokine expression are closely involved in the mechanisms of renal injury progression.

- ACE2 levels were significantly reduced in INS patients with proteinuria in comparison to those without proteinuria and urinary concentrations of this enzyme negatively correlated with 24-h urinary protein excretion.
- The chemokines MCP-1/CCL2 and IP-10/CXCL-10 may also contribute to proteinuria and to changes in RAS molecules in INS patients.
- The understanding on the interactions between RAS and immunoinflammatory molecules enables new strategies for diagnosis and therapeutic approach.

## Abbreviations

ACE: angiotensin converting enzyme
ACE2: angiotensin converting enzyme 2
Ang-(1-7): angiotensin-(1-7)
Ang II: angiotensin II
ARB: AT1 receptor blocker
AT1: angiotensin type 1
GFR: glomerular filtration rate
IFN-γ: interferon gamma
IL: interleukin
INS: idiopathic nephrotic syndrome
MCP-1/CCL2: monocyte chemoattractant protein-1
MIG/CXCL9: monokine induced by gamma interferon
NS: nephrotic syndrome
PNU: Pediatric Nephrology Unit
RANTES/CCL5: regulated on activation normal T cell expressed and secreted
IP-10/CXCL-10: induced protein 10
RAS: renin angiotensin system
SEM: standard error of the mean
TNF: tumor necrosis factor
